# Correction: Microfluidic devices powered by integrated elasto-magnetic pumps

**DOI:** 10.1039/d1lc90063c

**Published:** 2021-06-24

**Authors:** Jacob L. Binsley, Elizabeth L. Martin, Thomas O. Myers, Stefano Pagliara, Feodor Y. Ogrin

**Affiliations:** Department of Physics and Astronomy, University of Exeter Physics Building, Stocker Road Exeter EX4 4QL UK jb778@exeter.ac.uk +44 (0)1392 725018; Platform Kinetics Limited Pegholme, Wharfebank Mills Otley LS21 3JP UK; Department of Biosciences, University of Exeter, Living Systems Institute Stocker Road Exeter EX4 4QD UK

## Abstract

Correction for ‘Microfluidic devices powered by integrated elasto-magnetic pumps’ by Jacob L. Binsley *et al.*, *Lab Chip*, 2020, **20**, 4285–4295, DOI: 10.1039/D0LC00935K.

The authors regret that the following items are incorrect in the original article.

Firstly, the units of viscosity are incorrectly labelled throughout the paper (listed as kg ms^−1^ as opposed to kg m^−1^ s^−1^). This impacts Fig. 5–7.

**Fig. 5:**
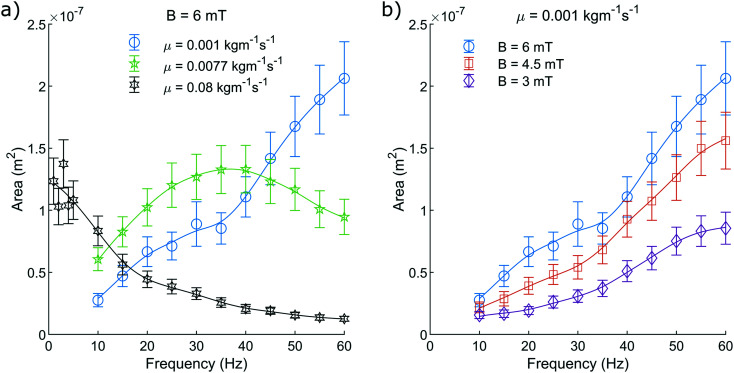
The pump motion as a function of frequency. a) Depicts the dependence on fluid viscosity with a constant field amplitude of 6 mT and b) depicts how the motion depends on field amplitude with a constant dynamic viscosity of 0.001 kg m^−1^ s^−1^. The blue curve is duplicated between both a) and b) for reference. The median area contained within the closed loop path traced by the non-reciprocal motion of the magnetic head of the pump, as suggested in Fig. 4, is shown. This is recorded as a function of driving frequency and repeated for a range of dynamic viscosities and driving field amplitudes. The plotted line is a simple smoothing spline between the data points to act as a visual guide.

**Fig. 6:**
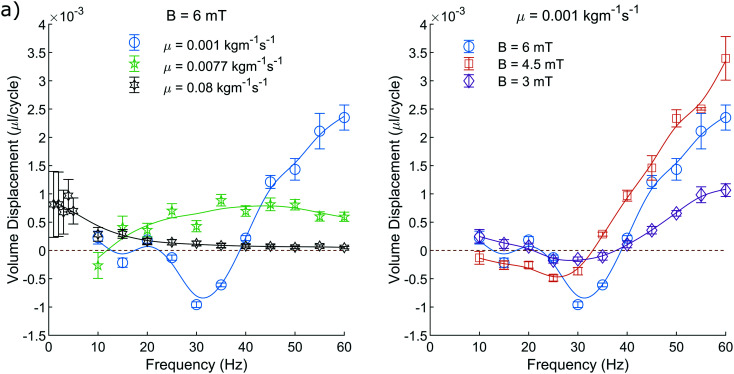
The net volume of fluid displaced per pump cycle. This consists of both the pump and recovery stroke. a) Depicts the displacement when varying the fluid viscosity at constant field amplitude of 6 mT. b) Depicts the displacement when varying the amplitude of the applied field at a constant fluid viscosity of 0.001 kg m^−1^ s^−1^. This is measured as described in section 2.4. The plotted line is a simple smoothing spline between the data points to act as a visual guide.

**Fig. 7:**
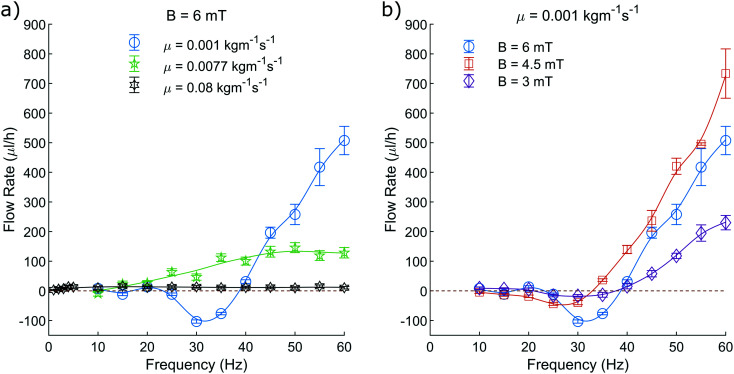
The average volumetric flow rates produced by this pumping system. a) Shows the volumetric flow rate when varying the fluid viscosity at constant field amplitude of 6 mT. b) Shows the volumetric flow rate when varying the amplitude of the applied field at a constant fluid viscosity of 0.001 kg m^−1^ s^−1^. The plotted line is a simple smoothing spline between the data points to act as a visual guide.

Additionally, in section 2.6, a constant ratio between *Q* and *v*_peak_ was found to be ∼775/2104, which is in fact a value taken from an intermediate step, which was also miscalculated. The correct ratio between *Q* and *v*_peak_ should be stated as ∼0.430 × 10^−6^. Due to the miscalculation included, the flow rates represented in the abstract and Fig. 6 and 7 are, in fact, ∼30.0% greater than originally stated. Since this scaling affects all flow rates proportionally, it does not change the shape of the plotted curves, and therefore does not alter the discussions or conclusions drawn from them.

Please find the corrected figures below.

The Royal Society of Chemistry apologises for these errors and any consequent inconvenience to authors and readers.

## Supplementary Material

